# Pregnancy outcome following opioid exposure: A cohort study

**DOI:** 10.1371/journal.pone.0219061

**Published:** 2019-07-01

**Authors:** Boris Fishman, Sharon Daniel, Gideon Koren, Eitan Lunenfeld, Amalia Levy

**Affiliations:** 1 Department of Public Health, Faculty of Health Sciences, Ben-Gurion University of the Negev, Beer-Sheva, Israel; 2 Department of Pediatrics, Soroka Medical Center, Beer-Sheva, Israel; 3 Faculty of Health Sciences, Ben-Gurion University of the Negev, Beer-Sheva, Israel; 4 Motherisk Israel, Tel Aviv, Israel; 5 Maccabi Health Services, Tel Aviv, Israel; 6 Department of Obstetrics and Gynecology, Soroka Medical Center, Beer-Sheva, Israel; Ohio State University, UNITED STATES

## Abstract

**Introduction:**

Opioids constitute a cornerstone of pain relief treatment. However, opioid safety during pregnancy has not been well established. Recent studies reported an association between in utero opioid exposure and spina bifida.

**Methods:**

In order to further evaluate the association of opioids exposure during pregnancy with adverse pregnancy outcomes, we conducted a large historical cohort by linking four databases: medications dispensations, births, pregnancy terminations for medical reasons and infant hospitalizations during the years of 1999–2009. Confounders that were controlled for included maternal age, ethnicity, maternal diabetes, smoking status, parity, obesity, year and folic acid intake. A secondary analysis for total major malformations and for spina bifida was performed using propensity score matching for first trimester exposure.

**Results:**

Of the 101,586 women included in the study, 3003 were dispensed opioids during the first trimester. Intrauterine exposure to opioids was not associated with overall major malformations (adjusted odds ratio (aOR) 0.97, 95% CI 0.83–1.13), cardiovascular malformations (aOR = 0.89, 95% CI 0.70–1.13) other malformations by systems or spina bifida in particular. However, the risk for spina bifida among newborns and abortuses who were exposed to codeine was four times higher than that of the unexposed (aOR = 4.42, 95% CI 1.60–12.23). This association remained significant in a secondary analysis using propensity score matching. Third trimester exposure to opioids was not associated with low birth weight (aOR = 1.08, 95% CI 0.77–1.52), perinatal death (aOR = 1.38, 95% CI 0.64–2.99) and other adverse pregnancy outcomes.

**Conclusions:**

These findings suggest that opioids exposure (as a homogenous group) is not a significant risk factor for overall major malformations. Exposure to codeine during the first trimester was found to be associated with increased risk of spina bifida. However, this finding was based on a small number of cases and need to be verified in future work.

## Introduction

Opioid medications are commonly used by the general population and by women of reproductive age, particularly in developed countries[1–3]. Indeed, it has been shown that each year, 27–39% of reproductive age women in the US fill opioid prescriptions[4]. Opioids are mostly indicated for the treatment of pain and codeine is also prescribed as antitussive treatment.

Opioids have been shown to cross many surface barriers, including the placenta, eventually reaching fetal circulation[5,6]. Despite the extensive use of opioids by pregnant women, however, few studies have addressed the safety of fetal exposure during the first trimester of pregnancy. Although most of these studies did not find any association between opioids and congenital malformations[7–11], two recent, large case control studies found first trimester exposure to opioids to be associated with heart and neural tube defects (NTDs)[12,13]. Of potential importance, none of the studies published so far have included pregnancy terminations for medical reasons in their cohorts.

Our objective was to further evaluate the risk of adverse pregnancy outcomes following opioids and specifically propoxyphene and codeine exposure, during the first and third trimesters of pregnancy in a large, cohort study.

## Materials and methods

### Study population

We conducted a historical cohort study that included all women between the ages of 15 and 49 who were insured by the Clalit Health Maintenance Organization (CHMO). The CHMO insures more than 70% of reproductive age women in the Beer-Sheva district of Israel, which had 664,000 inhabitants in 2013. No differences were found between the population insured by the CHMO and the population insured by other health organizations in the southern district of Israel[14]. Of the deliveries in this district, 98% take place at Soroka Medical Center (SMC), the main regional hospital and the only one in this district.

The women included in our study attended SMC between the years of 1999 and 2009 to give birth or to undergo pregnancy termination due to confirmed or suspected malformations in the fetus. The risk for major malformations was previously shown to be increased among multiple gestations compared with singleton pregnancies[15]. Therefore, those pregnancies were excluded from this study. We also excluded fetuses with chromosomal aberrations, fetuses exposed to folic acid antagonists or antiepileptic medications (e.g., methotrexate and valproic acid) during the first trimester of the pregnancy and pregnancies of women who were known to use illicit drugs in the present or in the past (based on self-report during their hospitalization for birth or pregnancy termination or social services report).

### Databases

To create the cohort, we combined four databases, three of which are computerized and based on information taken directly from original sources. The first computerized database is the SMC deliveries database, which contains information about all deliveries that took place at SMC, including maternal demographic information, parity, self-reported tobacco use during pregnancy, maternal medical conditions before and during pregnancy (e.g., pre-gestational diabetes mellitus and gestational diabetes mellitus), gestational age at delivery and delivery outcome. The second is the SMC pediatric hospitalizations database, which records information on all congenital malformations diagnosed up to one year of age and is encoded according to the international classifications of diseases, 9^th^ revision (ICD-9). The third electronically recorded database contains information on drug dispensations, including date of dispensation and Anatomical Therapeutic Chemical (ATC) classification. A fourth database on pregnancy termination performed due to confirmed or suspected malformations in the fetus was manually created using the registry of the Committee for Termination of Pregnancies at SMC. This database includes maternal demographic information, date of termination, pregnancy age at the time of termination, and diagnoses of fetal malformations. The datasets were established in 2011 and were encoded and linked by the personal identification number assigned to every patient at SMC. All pregnancy records were successfully linked with newborn and children's records. There was only one woman who had one record of a pregnancy in the cohort, in which we did not find any dispension of any drug in the medication database. Since the southern district population comprises mostly religious Bedouin and Jewish population, the prevalence of smoking in our data was relatively low.

### Study design

The exposed group included the newborns and fetuses of women to whom opioids were dispensed during the first 13 weeks of gestation. The prescribed opioids were: propoxyphene, codeine, tramadol, oxycodone and fentanyl. No other opioids were prescribed to the study’s participants. The first day of the last menstrual period was defined as the first day of pregnancy. The unexposed groups comprised the newborns and fetuses of all women who were not exposed to opioids during the first trimester. Because a relatively large number of exposures were of codeine (46%) and propoxyphene (49%) we performed sub-analyses for exposure to those specific medicines. Other opioids were not analyzed separately due to small number of exposures and a lack of statistical power.

We investigated the proportion of major malformations in newborns and fetuses after first trimester exposure to opioids and specifically to propoxyphene and codeine. We used the definition of major congenital malformations as defined by the Metropolitan Atlanta Congenital Defects Program of the Centers for Disease Control and Prevention[16–18]. In addition, we performed subclass analyses to investigate the risk of malformations by system and the specific malformations: central nervous system(CNS) malformations, including NTDs (ICD-9 codes: 740–742), NTDs (ICD-9 codes:740–741), cardiovascular system malformations (ICD-9 codes: 744–747), gastrointestinal tract system malformations (ICD-9 codes: 750–751), genitourinary system malformations (ICD-9 codes:751–752), musculoskeletal malformations (ICD-9 codes:753–754), spina bifida (SB) (ICD-9 code:741), anencephaly (ICD-9 code:740), and cleft palate and lip (ICD-9 code:749).

Furthermore, we investigated third trimester exposure to opioids in association with adverse birth outcomes. Those adverse outcomes might be associated with earlier gestational origin but third trimester in utero exposure to several drugs and chemicals was previously shown to affect fetal growth[19]. We investigated the risk among live neonates and stillbirths of the following adverse outcomes following third trimester (starting from week 29 of gestation) exposure to opioids and specifically to propoxyphene and codeine: perinatal death, low birth weight (<2500g), very low birth weight (<1500g), small for gestational age (SGA), and low Apgar score (<8) at 1 and 5 minutes after birth. We also analyzed the association between third trimester opioid exposure and neonatal abstinence syndrome (ICD9 code 779.5).

Malformations were detected in the neonatology unit by board-certified neonatologists and during hospitalizations of infants up to one year old in the SMC. For pregnancy terminations, malformations were diagnosed using ultrasound scans performed by gynecology and obstetrics physicians.

### Statistical analysis

We used the SPSS software, version 17, to perform the statistical analyses (IBM SPSS; Somers, NY). Maternal characteristics of the exposed and unexposed groups were compared using the chi-square test for categorical variables and Student’s t-test for continuous variables. We tested multivariable logistic-regression models to determine whether intrauterine first trimester exposure to opioids was independently associated with congenital major malformations, malformations by system and specific malformations. The models were adjusted for the following maternal demographic characteristics and other known risk factors for congenital malformations: maternal age (in months), ethnic group (Bedouin Muslim or Jewish), self-reported tobacco use during pregnancy, pre-gestational diabetes mellitus (DM), obesity, nulliparity (yes/no). In addition to the confounders listed above, the models for CNS malformations, including NTD, were also adjusted for folic acid intake during the period starting three months before pregnancy to the end of the first trimester. We tested additional models to address the association of propoxyphene or codeine specifically with malformations as described above. In addition, to further validate our results, we performed a sub-analysis by examining the risk for major congenital malformations by the number of prescriptions dispensed during the first trimester of pregnancy.

We also performed multivariable logistic regression analyses to evaluate the association between third trimester exposure and other adverse pregnancy outcomes as mentioned (e.g., low birth weight, perinatal death). In addition to the potential confounders used in the models for first trimester exposure, for the third trimester models, we adjusted for gestational DM, congenital malformations and lack of pregnancy care, which was defined as three or fewer physician visits during pregnancy.

Propensity score matching (R, the MatchIt package) was performed in order to address possible selection bias i.e, the population of exposed pregnancies is different in characteristics from the non-exposed pregnancies population, such that exposed pregnancies are only compared with a group of non-exposed pregnancies that is similar in characteristics. This analysis was conducted for total major malformations following first trimester exposures to opioids overall and separately for codeine and propoxyphene. The variables that were used to create the propensity model were maternal age, ethnic group, tobacco use, diabetes mellitus (DM), obesity, nulliparity and the use of folic acid supplements. The matching was performed such that the propensity for drug exposure for every exposed pregnancy was as close as 0.1 standard deviations from the propensity of the matched unexposed pregnancies. The odds ratio (OR) and 95% confidence interval (CI) for total major malformations and for specific malformations found to be significant in preliminary analysis were calculated.

This study was approved by the local institutional ethics committee at Soroka Medical Center in accordance with the principles of the Declaration of Helsinki.

The SMC research ethics committee waived the requirement for informed consent because the data were obtained anonymously from medical files with no participation of patients.

## Results

There were 100,491 singleton births and 1095 pregnancy terminations from 1999 to 2009 in SMC to mothers who did not use folic acid antagonists or anticonvulsants during the first trimester of their pregnancies. Overall, 3003 (3%) fetuses were exposed to at least one opioid medication during the first trimester, corresponding to 2,957 (2.9%) among the 100,491 live born infants and 46 (4.2%) of the 1095 pregnancy terminations. 1480 (1.5%) fetuses were exposed solely to propoxyphene and 1390 (1.4%) were exposed only to codeine without being exposed to other opioids during that period of the pregnancy, 50 pregnancies were exposed to tramadol, 7 to oxycodone and 2 to fentanyl. a total of 72 pregnancies were exposed to more than one opioid medication during the first trimester. A comparison of maternal characteristics between the exposed and unexposed groups is presented in Table 1.

**Table 1:** Comparison of maternal characteristics of women unexposed to any of the opioids during the first trimester of pregnancy to women exposed to any of the opioids and to codeine or propoxyphene alone.

	Not exposed to any of the opioids n = 98,583 (97%)	Exposed to any opioid n = 3003 (3%)	Exposed to Codeine n = 1390 (1.4%)	Exposed to Propoxyphene n = 1480 (1.5%)
Age (mean±S.D)	28.6±5.8	30.26±6.1	30.25±6.0	30.08±6.1
Ethnicity (Bedouins)	63,298 (64.2%)	2111 (70.3%)	822 (59.2%)	1203 (81.3%)
Pre-gestational diabetes	994 (1%)	51 (1.7%)	23 (1.7%)	25 (1.7%)
Smoking during pregnancy	1910 (1.9%)	87 (2.9%)	49 (3.5%)	35 (2.4%)
Maternal obesity	273 (0.3%)	4 (0.1%)	2 (0.1%)	2(0.1%)
Nulliparity (yes/no)	21,622 (22%)	419 (14%)	219 (15.8%)	183 (12.4%)
Folic acid intake	24,919 (25.3%)	1088 (36.2%)	512 (36.8%)	528 (35.7%)

Abbreviations: SD, standard deviation

The unadjusted and adjusted risks for total congenital malformations and congenital malformations by system following first trimester exposure to opioids as a group and specifically to propoxyphene and codeine are presented in Table 2 and Table 3, respectively. The proportion of total major malformations in the total opioids group was 5.9% (178 out of 3003) compared to 6.1% (5986 of 98,853) in the unexposed group (crude odds ratio 0.93, 95% CI 0.73–1.19; aOR 0.95, 95% CI 0.82–1.11). No significant association was found between in utero propoxyphene and codeine exposure and major malformations (aOR 0.91, 95% CI 0.72–1.15 and aOR 0.95, 95% CI 0.77–1.18, respectively). In addition, no significant association was found between first trimester exposure to total opioids or to propoxyphene or to codeine specifically and total malformations and malformations of the cardiovascular, CNS, NTDs, genito-urinary, musculoskeletal, gastrointestinal systems or malformations such as cleft lip and palate (Table 2 and Table 3).

**Table 2:** Unadjusted and adjusted risk (odds ratios (OR) and 95% confidence intervals (95% CI)) for congenital malformations after intrauterine exposure to opioids during the first trimester of pregnancy compared to unexposed.

Major Malformations	Unexposed n = 98,583 (97%) n (%)	Opioids Exposed n = 3003 (3%) n (%)	Unadjusted OR (95% CI)	*Adjusted OR (95% CI)	P
Total	5986 (6.1)	178 (5.9)	0.93 (0.73–1.19)	0.95 (0.82–1.11)	0.554
CVS	2462 (2.5)	70 (2.3)	0.90 (0.71–1.15)	0.89 (0.70–1.13)	0.326
CNS (NTD included)	821 (0.8)	24 (0.8)	0.96 (0.64–1.44)	0.90 (0.59–1.37)	0.621
NTDs	162 (0.2)	5(0.2)	1.01 (0.42–2.47)	1.13 (0.46–2.76)	0.793
Spina bifida	72 (0.1)	4 (0.1)	1.82 (0.67–5.00)	1.82 (0.66–5.03)	0.246
Anencephaly	72 (0.1)	1 (0.01)	0.81 (0.52–1.27)	0.57 (0.08–4.12)	0.577
Genitourinary	805 (0.8)	20 (0.7)	0.81 (0.52–1.27)	0.80 (0.51–1.24)	0.315
Musculoskeletal	1483 (1.5)	51 (1.7)	1.13 (0.85–1.50)	1.14 (0.86–1.51)	0.369
Gastrointestinal	308 (0.3)	11 (0.4)	1.17 (0.64–2.14)	1.08 (0.59–1.98)	0.795
Cleft lip/palate	134 (0.1)	2 (0.1)	0.49 (0.12–1.98)	0.47 (0.12–1.91)	0.293

Abbreviations: OR, odds ratio; CVS, cardiovascular; CNS, central nervous system; NTDs, neural tube defects

*Adjusted for: maternal age (in months), ethnic group (Bedouin Muslim or Jewish), self-reported tobacco use during pregnancy, pre-gestational diabetes mellitus, maternal obesity, nulliparity (yes/no) and folic acid intake (for models of CNS malformations, including NTD).

**Table 3:** Odds ratios for congenital malformations after intrauterine exposure to codeine or propoxyphene during the first trimester of pregnancy compared to unexposed.

Major malformations	Unexposed n = 98,583 (97% (%)n	Codeine exposed n = 1390 (1.4%) n (%)	*Adjusted OR (95% CI)	P	Propoxyphene exposed n = 1480 (1.5%) n (%)	*Adjusted OR (95% CI)	P
Total	5986 (6.1)	75 (5.4)	0.91 (0.72–1.15)	0.49	90 (6.1)	0.95 (0.77–1.18)	0.75
CVS	2462 (2.5)	31 (2.2)	0.90 (0.63–1.28)	0.61	36 (2.4)	0.89 (0.63–1.24)	0.53
CNS (NTD included)	821 (0.8)	16 (1.2)	1.46 (0.89–2.41)	0.13	6 (0.4)	0.45 (0.200–1.01)	0.05
NTDs	162 (0.2)	4 (0.3)	2.04 (0.75–5.53)	0.16	1 (0.1)	0.45 (0.06–3.21)	0.42
Spina bifida	72 (0.1)	4 (0.3)	4.42 (1.60–12.23)	<0.01	0	0	
Anencephaly	72 (0.1)	0	0		1 (0.1)	1.16 (0.16–8.42)	0.88
Genitourinary	805 (0.8)	6 (0.4)	0.53 (0.24–1.18)	0.13	13 (0.9)	1.02 (0.59–1.78)	0.89
Musculoskeletal	1483 (1.5)	20 (1.4)	1.00 (0.64–1.56)	0.96	25 (1.7)	1.09 (0.73–1.63)	0.63
Gastrointestinal	308 (0.3)	3 (0.2)	0.67 (0.21–2.09)	0.49	8 (0.5)	1.52 (0.75–3.09)	0.24
Cleft lip/palate	134 (0.1)	1 (0.1)	0.56 (0.08–4.04)	0.55	1 (0.1)	0.44 (0.06–3.18)	0.41

Abbreviations: OR, odds ratio; CVS, cardiovascular; CNS, central nervous system; NTDs, neural tube defects

*Adjusted for: maternal age (in months), ethnic group (Bedouin Muslim or Jewish), self-reported tobacco use during pregnancy, pre-gestational diabetes mellitus, maternal obesity, nulliparity (yes/no) and folic acid intake (for models of CNS malformations, including NTD).

An association was found between first trimester exposure to codeine and SB (crude OR 3.95, 95% CI 1.44–10.822; adjusted OR 4.42, 95% CI 1.60–12.23). However, no association was found in terms of exposure to total opioids or to propoxyphene.

### Sensitivity analyses

In a sub-analysis, the risk for major congenital malformations was not increased among pregnancies with increasing number of prescriptions for opioids dispensed during the first trimester of pregnancy (aOR = 0.8 95% CI 0.62–1.01, aOR = 0.98, 95%CI 0.34–2.45 and aOR = 0.98, 95%CI 0.13–7.51) for pregnancies with one prescription, pregnancies with two prescriptions and pregnancies with three or more prescriptions during the first trimester, respectively).

In a secondary analysis using propensity score matching we matched pregnancies exposed to opioids during the first trimester of pregnancy with unexposed pregnancies at the ratio of 1:12. A total of 2999 pregnancies exposed to opioids overall were matched with 35,928 unexposed pregnancies, 1479 pregnancies exposed to propoxyphene were matched with 17,732 unexposed pregnancies and 1386 pregnancies exposed to codeine were matched with 16,590 unexposed pregnancies. In this analysis, no increased risk for total major malformations was found following first trimester exposure to opioids overall (OR 0.95, 95% CI 0.72–1.17), and specifically to propoxyphene (OR 0.96, 95% CI 0.76–1.19) and codeine (OR 0.92, 95% CI 0.72–1.17). In contrast, first trimester exposure to codeine was found significantly associated with SB (OR 3.69, 95% CI 1.04–10.45).

Analyses of third trimester exposure did not find any association between total opioids, propxyphene or codeine exposure with other adverse pregnancy outcomes (Table 4). There were 15 neonates that were diagnosed with neonatal abstinence syndrome after birth. None of those 15 neonates was exposed to opioids during the third trimester of pregnancy

**Table 4:** Odds ratios for adverse pregnancy outcomes (other than malformations) following third trimester intrauterine exposure to opioids, and specifically to codeine or to propoxyphene alone, compared to unexposed.

Adverse outcome	Unexposed n = 98,849 (98.4%) n (%)	Opioids exposed n = 1638 (1.6%) n (%)	*Adjusted OR (95% CI)	Codeine exposed n = 858 (0.9%) n (%)	*Adjusted OR (95% CI)	Propoxyphene exposed n = 733 (0.7%) n (%)	*Adjusted OR (95% CI)
Birth weight<2500 gr	8194 (8.3)	97 (6.0)	1 (0.79–1.26)	48 (5.6)	0.93 (0.67–1.29)	45 (6.1)	1.08 (0.77–1.52)
Birth weight<1500 gr	1286 (1.3)	5 (0.3)	1.20 (0.43–3.36)	2 (0.2)	1.22 (0.28–5.37)	2 (0.3)	1.03 (0.19–5.59)
Perinatal death	1209 (1.2)	11 (0.7)	1.0 (0.54–1.83)	4 (0.5)	0.73 (0.27–1.96)	7 (1)	1.38 (0.64–2.99)
Apgar score<8 at 1 minute after birth	5617 (5.8)	87 (5.4)	0.98 (0.79–1.22)	46 (5.4)	1.05 (0.77–1.42)	36 (5.0)	0.85 (0.60–1.19)
Apgar score<8 at 5 minutes after birth	983 (1.0)	15 (0.9)	1.29 (0.77–2.16)	8 (0.9)	1.38 (0.68–2.79)	5 (0.7)	0.91 (0.37–2.20)
Small for gestational age	5730 (5.7)	73 (4.4)	0.82 (0.65–1.04)	41 (4.8)	0.85 (0.62–1.17)	30 (4.1)	0.78 (0.54–1.13)

*Adjusted for: maternal age (in months), ethnic group (Bedouin Muslim or Jewish), self-reported tobacco use during pregnancy, pre-gestational or gestational diabetes mellitus, maternal obesity, nulliparity (yes/no), congenital malformations and lack of pregnancy care.

## Discussion

Opioids are one of the cornerstones of analgesia treatment in the general population and in pregnant women in particular. With half of all pregnancies unplanned, a potentially high proportion of pregnant women are exposed to this class of drugs. Our large historical study failed to show an association between exposure to opioids as a group in the first trimester and overall major malformations proportion or malformations by system. Separate analyses of codeine and propoxyphene exposure also failed to demonstrate most of these associations. However, exposure to codeine during the first 13 weeks of pregnancy was associated with SB (aOR 4.42, 95% CI, 1.60–12.23). Furthermore, no increased risk for major congenital malformations was found among pregnancies with increasing number of prescriptions dispensed during the first trimester of pregnancy. Our study did not show an association between exposure opioids during the third trimester of pregnancy and other adverse pregnancy outcomes, such as perinatal death.

Our study found a higher proportion of major malformations than the proportions that have been reported in previous reports from around the world [20] with several possible explanations for this finding. Our cohort also included pregnancy terminations done for medical reasons. Most of the pregnancies of in our study were of women of Bedouin ethnicity, an ethnic group that mostly dwell in southern Israel. To the best of our knowledge no differences exist in the proportion of opioid use between Bedouins and the rest of the Israeli population. Nevertheless, Bedouins are from the lowest socio-economic status in Israel (according to the Israeli Central Bureau of Statistics) which is independently associated with increased risk for major congenital malformations[21,22]. Furthermore, Bedouins are known to have higher proportion of congenital malformations compared to Jews, due, in part, to the high prevalence of consanguinity [23,24].

Anti folic acid drugs are among the more common drugs known to be causing congenital malformations (Anti-epileptic medications in particular) in women of reproductive age[25], and therefore, pregnancies exposed to anti-folates were excluded from this study.

The findings of our study are consistent with the results of most previous studies addressing the safety of prenatal exposure to opioid medications and to codeine in particular[7–11]. A study by Kallen et al.[9] based on the Swedish Medical Registry did not find opioids, and specifically, codeine and dextropropoxyphene, to be associated with congenital malformations, including cardiac malformations. Similar to our results, the case control studies conducted by Broussard et al.[12] and Yazdy et al.[13] found an association between exposure to opioids and SB (unadjusted OR 2.0, 95% CI 1.3–3.2; aOR 2.2, 95% CI 1.1–4.1, respectively).

SB, one of the most prevalent NTDs, has a marked impact on the life of the newborn[26]. It is estimated that more than 70% of SB cases can be prevented by increasing maternal folic acid consumption[26]. Among the most studied teratogens believed to cause SB are anticonvulsants[27], but other medications[12,13] have also been suggested to cause this malformation. Previous animal studies detected endogenous opioid growth receptors in the CNS that may affect DNA synthesis in early fetal developmental stages[28,29] while others showed that in utero opioid exposure caused delayed development of the spinal cord[30,31], possibly due to increased neuroblast apoptosis[32]. These findings suggest a biological mechanism for the epidemiological association found in our study between codeine exposure during the first trimester of pregnancy and SB.

### Limitations and strengths

A notable limitation of our study is that the databases used to build our cohort contained information about the dispensation of opioid medications to pregnant women, but we have no direct knowledge about patient adherence to the recommended treatment. However, other studies have shown that computerized databases of drug dispensation are highly correlated with drug use by the general population[33–35] and by pregnant women in particular[36]. Prescription records were also found to be good source of data to study the association between drugs and congenital malformations[37]. To evaluate adherence to treatment, we compared the proportion of folic acid dispensation detected in our study to the proportion of folic acid consumption as reported by the Israeli Health Ministry[38] and found a similar proportions between the two. Another possible limitation is the misclassification of women as unexposed for those who have used opioids that were purchased prior to the examined period. Furthermore, the diagnosis of obesity was documented by the gynecologist on admission, hence, the proportion of obesity in our study is an underestimation of the proportion of obesity in the population. Nonetheless, the prevalence of adult obesity in Israel (20%) is lower compared with the prevalence in North America (36%)[39,40]. This study did not contain data on spontaneous abortions. Moreover, a potential association between exposure to opioids and pregnancy loss could potentially underestimate the association between opioid exposure and major malformations because fetuses might not survive long enough to be assessed for major malformations. Last, the opioids examined in this study are indicated for pain relief and for antitussive treatment. However, data regarding the specific indication of use was lacking. Nonetheless, we did not detect an association with adverse pregnancy outcomes, therefore the possibility of an indication bias is negligible.

Our study has several strengths. The study cohort was derived from SMC, the only hospital in the southern district of Israel, where practically all the births in this district take place. We adjusted our models for known risk factors for congenital malformations. Furthermore, this study included data regarding major malformations diagnosed on pregnancies that were terminated for a suspected malformation in the fetus. The inclusion of those observations was previously proved to prevent a bias towards the null hypothesis [41]. In addition, we excluded from our cohort fetuses and abortuses diagnosed with chromosomal abnormalities and those exposed in utero to folic acid antagonists or antiepileptic drugs, as these have been shown to increase the risk for major congenital malformations, particularly NTDs[25,26]. Finally, in order to overcome a possible selection bias regarding the exposure itself we performed a secondary analysis using propensity score matching for total major malformations following first trimester exposure to opioids overall and to propoxyphene and codeine specifically. Furthermore, a secondary analysis was also performed for the association between exposure to codeine and SB. Similar results were found. Although we tested the associations between opioids and various groups of major malformations, our results were not corrected for the number of comparisons. The p-value for the association between propoxyphene and Spina Bifida was lower than 0.001, therefore it is likely to remain significant after adjustment for multiple comparisons.

In conclusion, opioid medications as a group do not appear to be associated with increased risk of major malformations, malformations by systems, or specific malformations. Codeine and propoxyphene exposures were also not associated with total major malformations or with malformations by system. Our study supports previous studies suggesting an association between first trimester in utero codeine exposure and SB. However, the small number of cases among the exposed group in our study dictates the need for further research to clarify this association.
